# Supplementary material for: Causes and consequences of sex-chromosome turnovers in Diptera
Source: Evol Lett. 2026 Mar 12;10(3):253–68. doi: 10.1093/evlett/qrag003 (PMC13231274; doi:10.1093/evlett/qrag003)
Supplement: qrag003_Supplemental_Files [file qrag003_supplemental_files.zip › Supplement.Updated.23Dec25.pdf]

Supplementary Materials

Supplementary Figures

Figure S1: Graphical representation of *D. melanogaster genes* in chromosomes (only one species per family is represented).

Figure S2: Heatmap of Muller elements enrichment in *Bradysia coprophila* and enrichment of *B. coprophila* chromosomes in *Bibio marce*.

Figure S3: Fusions in the section Schizophora.

Figure S4: The X chromosome composition of Conopidae species and fusions.

Figure S5: Schizophora have increased turnover rates and a smaller proportion of genes on element F.

Figure S6: Fewer element F genes are recovered in Muscidae genomes (circles) than in other dipteran genomes (triangles) once divergence has been accounted for.

Figure S7: Expected and observed gene movements for Schizophora species which have co-opted a large Muller element as their X chromosome after turnover.

Figure S8: *D. melanogaster* ovary expression of homologs inferred to have moved chromosomes.

Figure S9: *D. melanogaster* testis expression of homologs inferred to have moved chromosomes, using ModEncode data obtained from mated 4-day old males.

Figure S10: *D. melanogaster* ovary expression of homologs inferred to have moved chromosomes, using ModEncode data obtained from mated 4-day old females.

Figure S11: *D. melanogaster* ovary expression of homologs inferred to have moved chromosomes, using ModEncode data obtained from virgin 4-day old females.

Figure S12: *D. melanogaster* female carcass expression of homologs inferred to have moved chromosomes.

Figure S13: *D. melanogaster* male carcass expression of homologs inferred to have moved chromosomes.

Figure S14: *D. melanogaster* female head expression of homologs inferred to have moved chromosomes.

Figure S15: *D. melanogaster* male head expression of homologs inferred to have moved chromosomes.

Figure S16: StringDB functional enrichment of genes that moved out of the X.

Supplementary Tables (https://doi.org/10.15479/AT-ISTA-20833)

Table S1: List of analysed genomes and corresponding citations.

Table S2: Enrichment of ME acting as the X.

Table S3: The proportion of mapped genes found on the chromosome homologous to element F.

Table S4: Protein annotations used for our Diptera-branched tree.

Table S5: The fate of genes ancestrally located on the X of Calyptratae.

Table S6: Number of gene movements.

Table S7: GO enrichment amount genes that moved out of the X in Diopsidae.

Table S8: GO enrichment amount genes that moved out of the X in Clusiidae.

Table S9: GO enrichment amount genes that moved out of the X in Conopidae (*Thecophora atra*).
Table S10: GO enrichment amount genes that moved out of the X in Drosophilidae.
Table S11: GO enrichment amount genes that moved between autosomes in Hippoboscidae.
Table S12: GO enrichment amount genes that moved between autosomes in Drosophilidae.

Supplementary Datasets (https://doi.org/10.15479/AT-ISTA-20780)
Supplementary Dataset 1: Heatmaps for enrichment of Muller elements in outgroups.
Supplementary Dataset 2: Graphical representation of the location of *Drosophila melanogaster* genes colored according to their original Muller element in each outgroup genome.
Supplementary Dataset 3: Gene counts. Number of *D. melanogaster* genes assigned to each chromosome in outgroup species.
Supplementary Dataset 4: Number and proportion of genes mapped to chromosomes homologous to element F.
Supplementary Dataset 5: GC content of all species with an X.
Supplementary Dataset 6: Out-of-X-FlyAtlas2
Supplementary Dataset 7: *Drosophila melanogaster* genes and their assigned chromosomal location in each outgroup genome.

Supplementary Figures

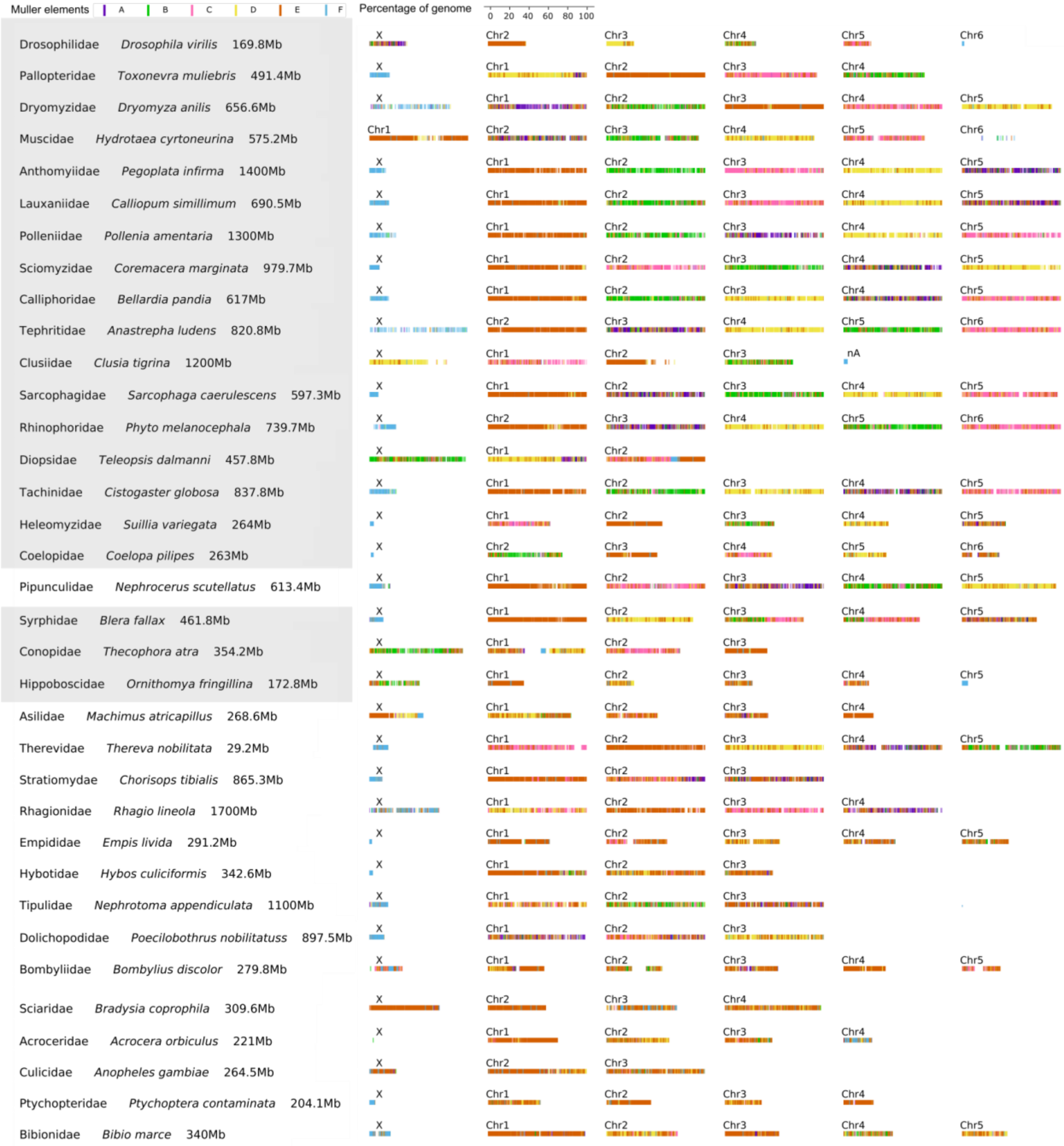

Figure S1: Graphical representation of *D. melanogaster genes* in chromosomes (only one species per family is represented). Each chromosome in the species has been normalized by the size of the corresponding genome. Names highlighted in gray correspond to flies in the Shizophora group.

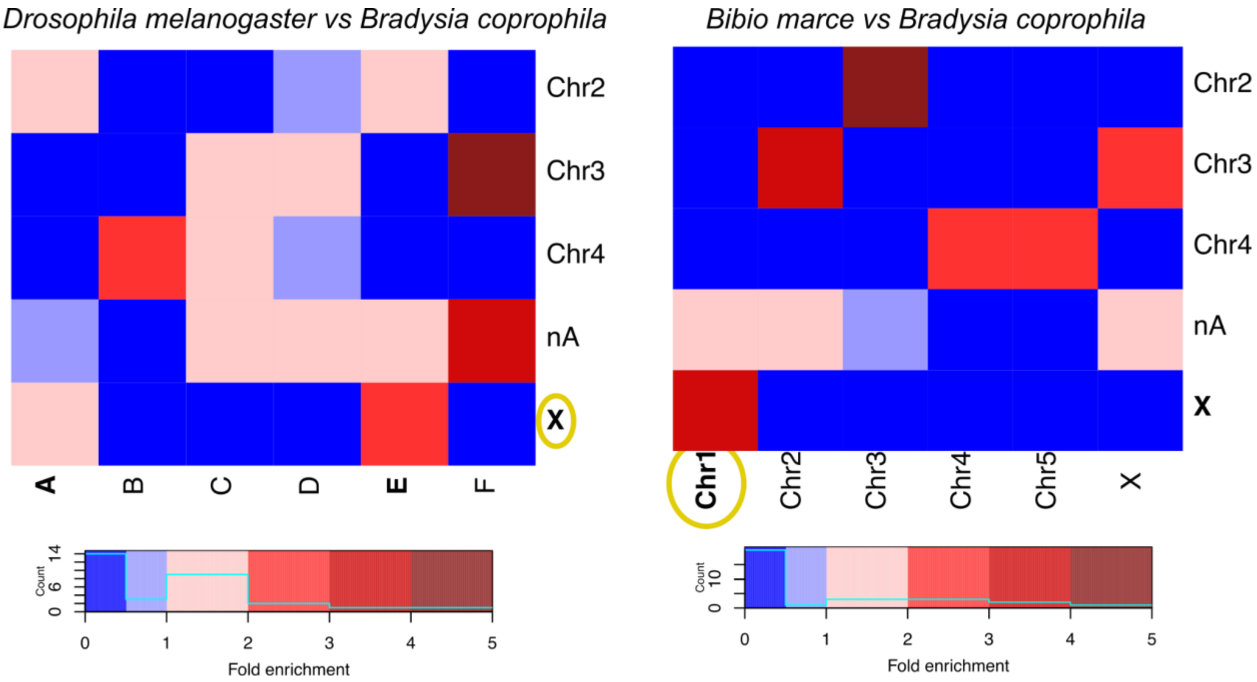

Figure S2: Heatmap showing the enrichment of Muller elements (x-axis) in the chromosomes of *Bradysia coprophila* (y-axis) (left) and the enrichment of *B. coprophila* chromosomes (x-axis) in the *Bibio marce* chromosomes (right). The X (elements A and E) in *B. coprophila* correspond to an autosome (Chr1) in the sister family Bibionidae (*Bibio marce)*, showing a simple turnover event.

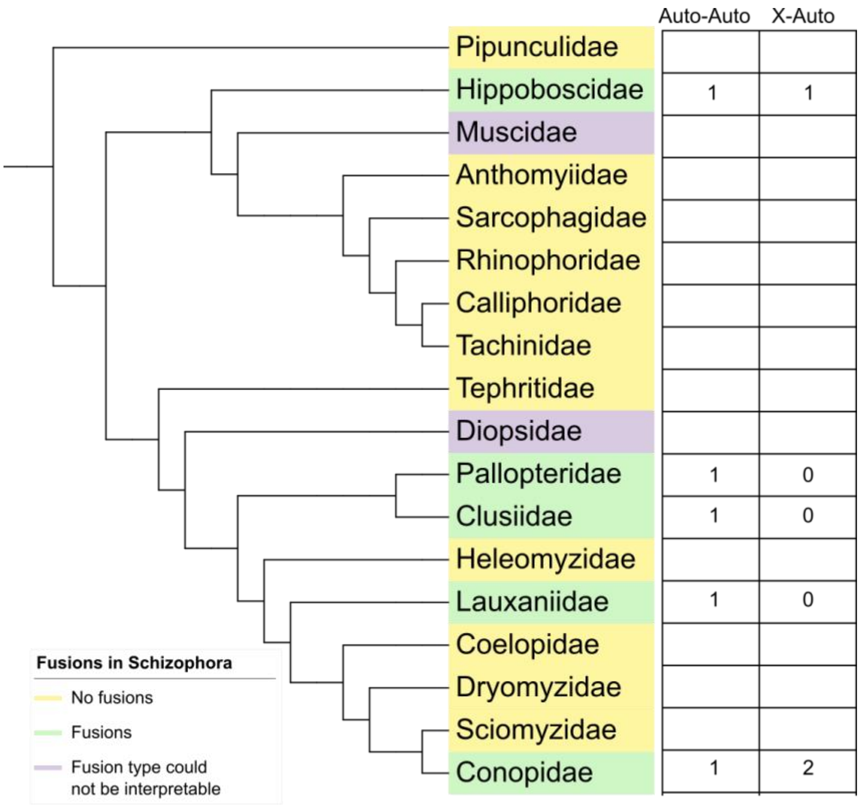

Figure S3: Fusions in the section Schizophora (where at least one chromosome-level assembly was available). Fusion type was not interpretable in Muscidae because X chromosomes are not characterized.

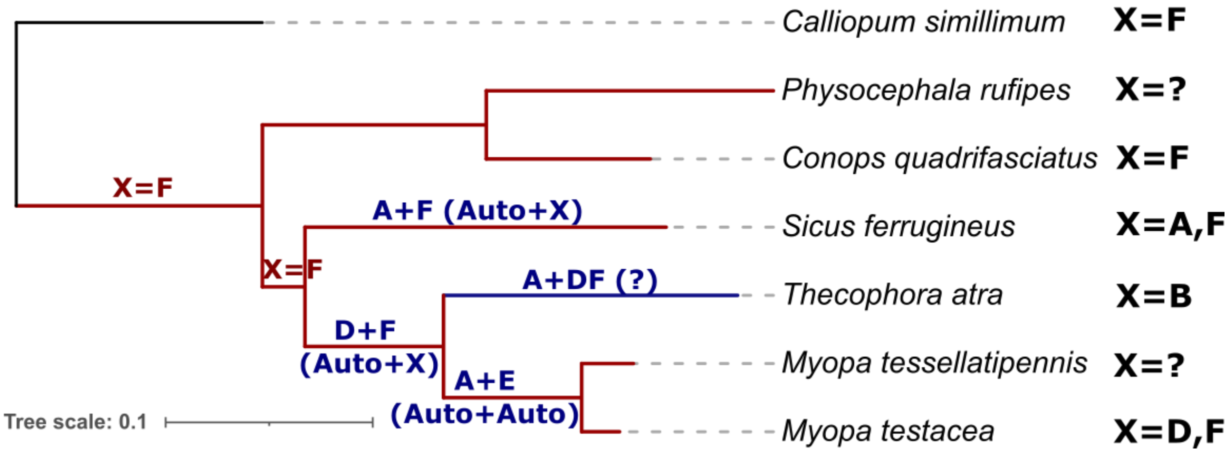

Figure S4: The X chromosome composition of Conopidae species and fusions involving the X and the autosomes. The red branches indicate that F has been maintained as the X, or even if another element was fused to it. The blue branch represents a turnover event.

A

Model	Log likelihood	P-value
ER-S	-23.0646	0.003494
ER-M	-18.7998	
ARD-S	-21.9376	0.007308
ARD-M	-17.0188	

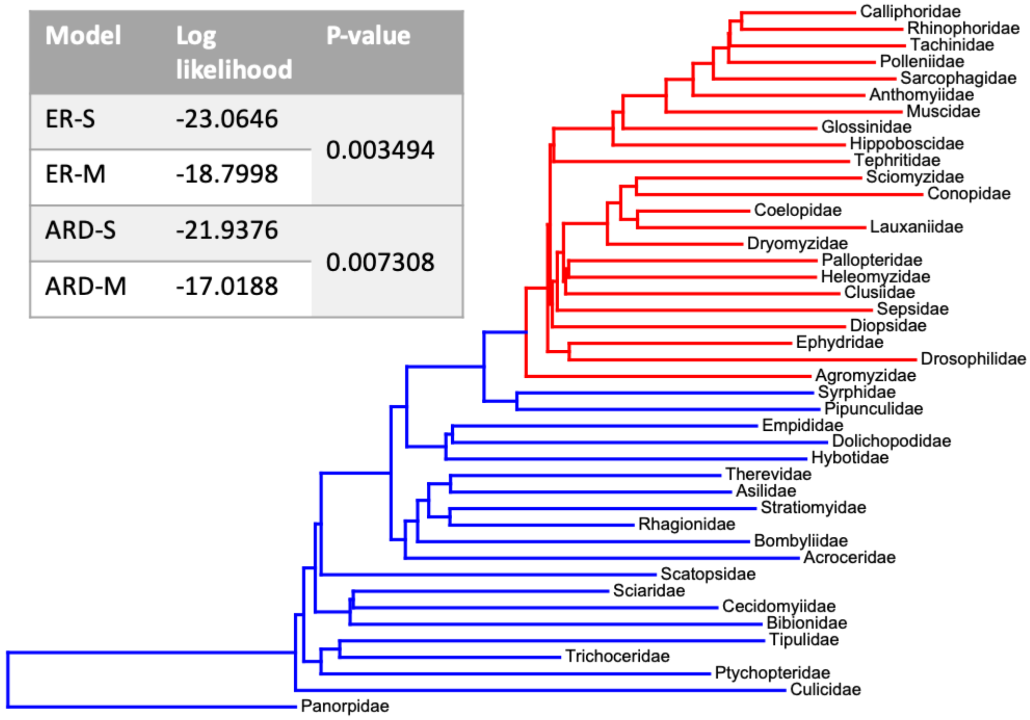

B

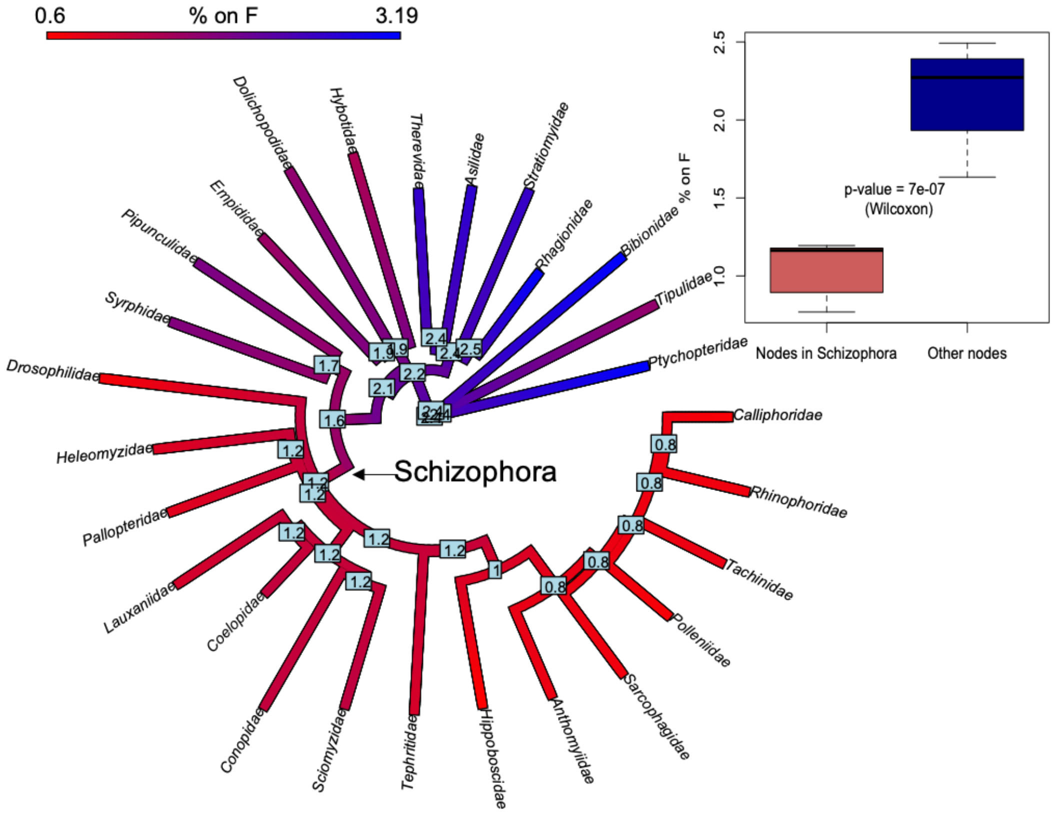

Figure S5: Schizophora have increased turnover rates and a smaller proportion of genes on element F. Full legend on the next page.

Figure S5 (continued): Panel A: Comparison of models with and without different turnover rates. The tree was split between Schizophora (red) and other branches (blue). The Phytools functions fitMk and fitmultiMk were used to compare the log-likelihood of models that assume a single turnover rate for the whole tree (ER-S and ARD-S) to models that assume two different turnover rates for the red and blue parts of the phylogeney (ER-M and ARD-M). ER assumes turnover and turnover reversal happen at equal rates, while ARD allows for asymmetric turnover and reveral rates. P-values were obtained with likelihood ratio tests. B. Ancestral reconstruction of the percentage of mapped genes located on element F throughout the dipteran phylogeny. Families for which only scaffold level assemblies were available, or where element F was fused to one of the large chromosomes, were excluded from this analysis (as it was not possible to infer the % of genes on F). Ancestral states were inferred with the Phytools function fastAnc. The labels at internal nodes show the inferred ancestral values for those nodes.

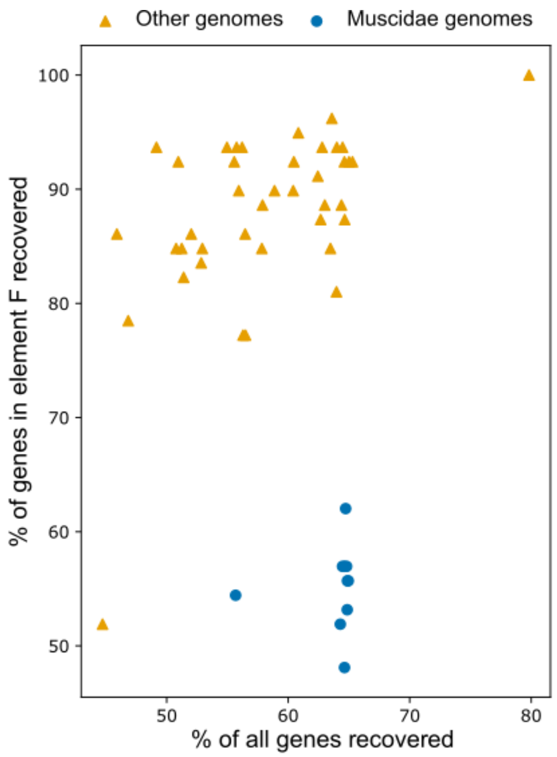

Figure S6: Fewer element F genes are recovered in Muscidae genomes (circles) than in other dipteran genomes (triangles) once divergence has been accounted for. The percentages refer to the percentage of *D. melanogaster* genes for which a mapping location was inferred in each of the diptera genomes, either when all genes are considered (x-axis) or when only element F genes are considered (y-axis).

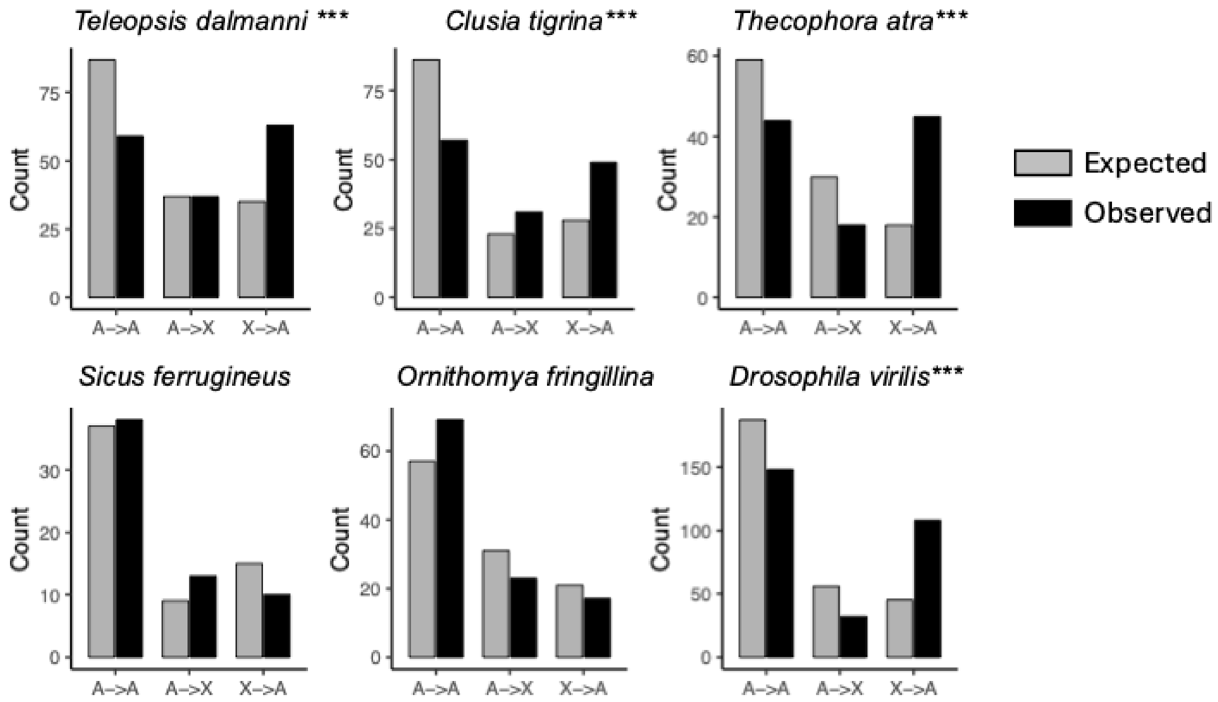

Figure S7. Expected (grey) and observed (black) gene movements for Schizophora species which have co-opted a large Muller element as their X chromosome after turnover. X->Auto refers to genes that moved out of the X, Auto->X refers to genes that moved into the X, Auto->Auto refers to genes that moved between autosomes. Species with significant deviations between the observed and expected number of gene movements are denoted with asterisks (p-values according to Chi-square tests: * for p<0.05, ** for p<0.01, *** for p<0.001).

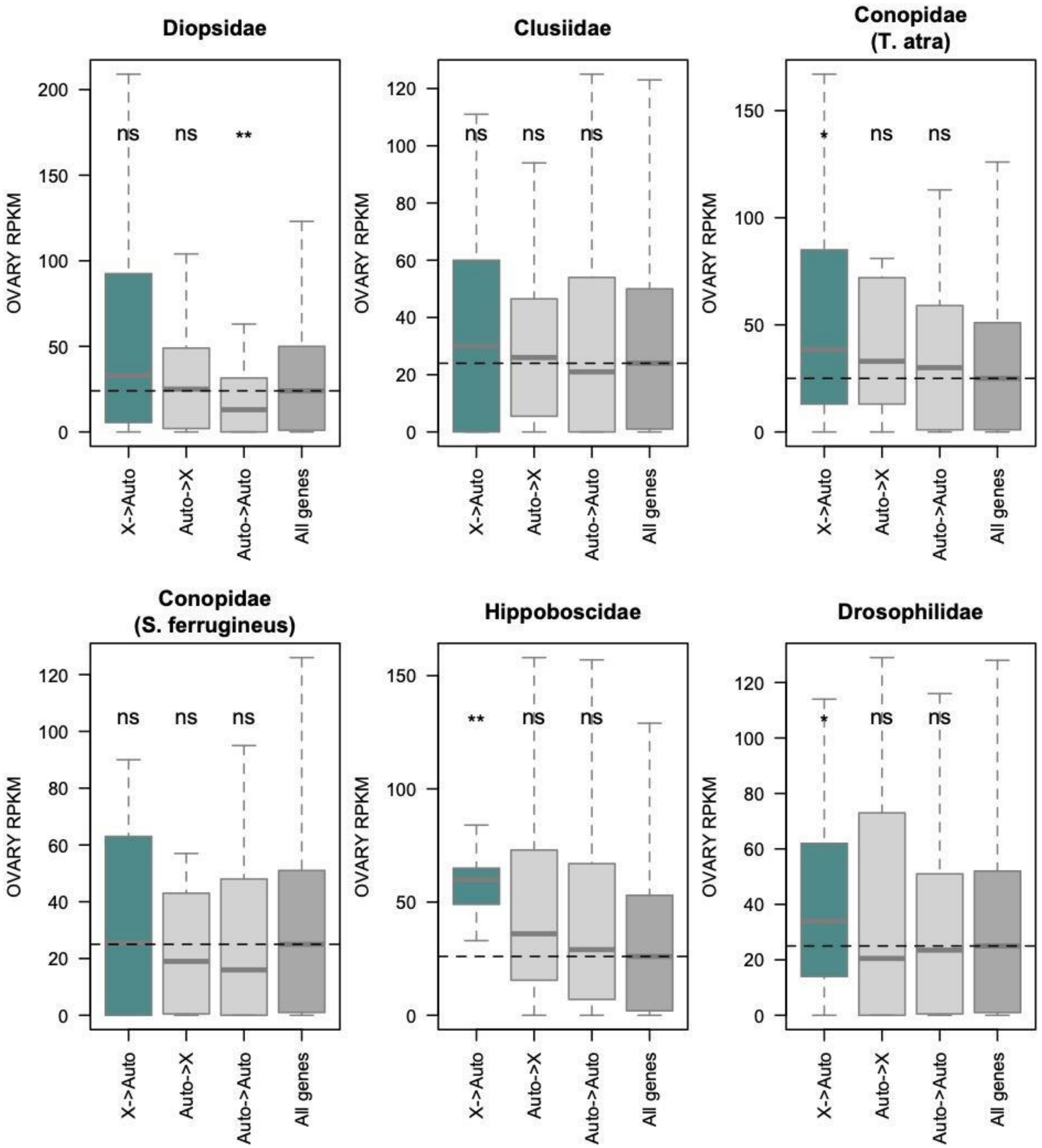

Figure S8: *D. melanogaster* ovary expression of homologs inferred to have moved chromosomes, and that of all mapped genes, for each of the trios (named after the focal lineage under study). X->Auto refers to genes that moved out of the X, Auto->X refers to genes that moved into the X, Auto->Auto refers to genes that moved between autosomes, and All genes refers to all mapped genes in each trio. Significance was assessed with Wilcoxon tests (* for p<0.05, ** for p<0.01, *** for p<0.001, ns for non significant).

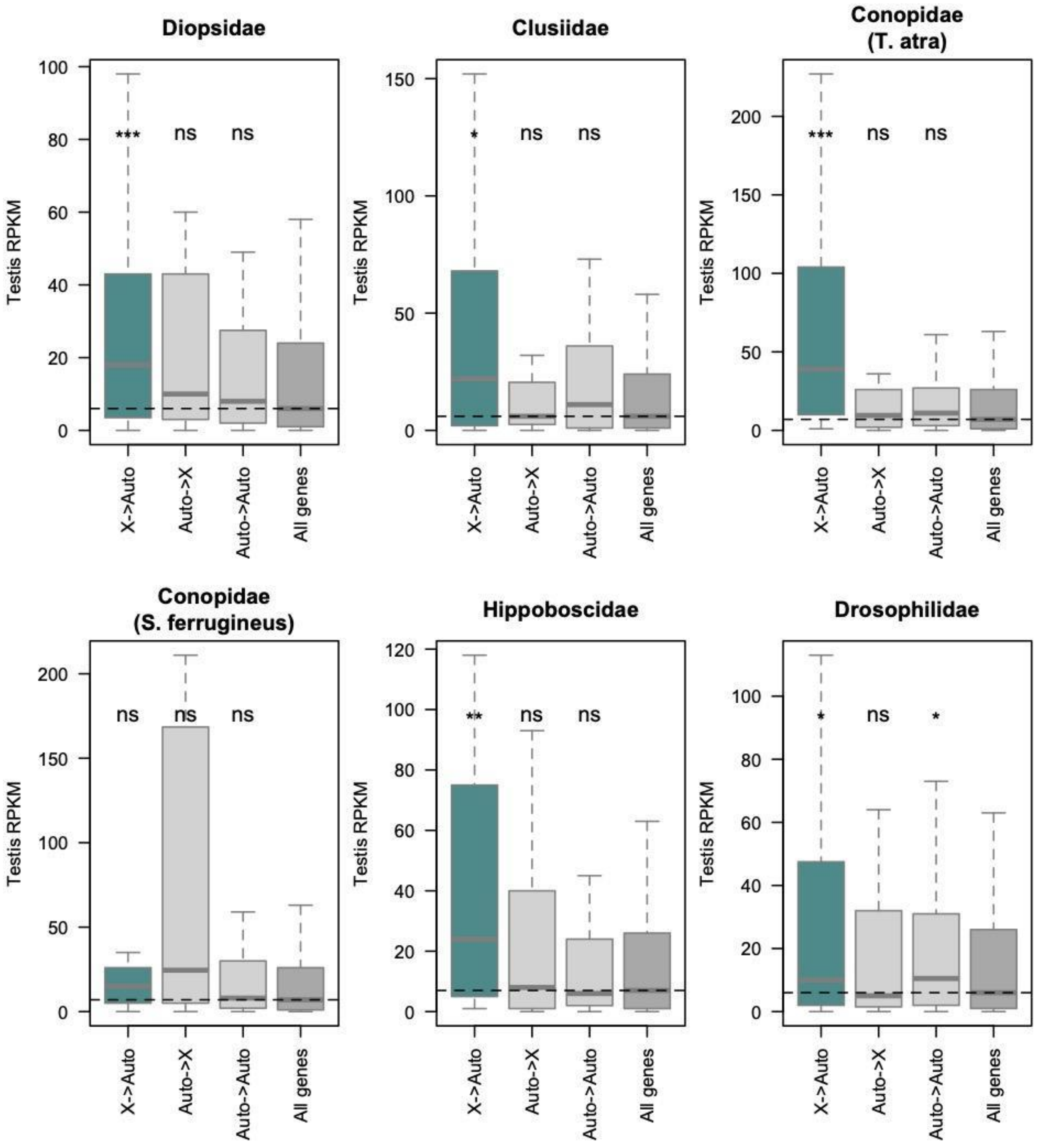

Figure S9: *D. melanogaster* testis expression of homologs inferred to have moved chromosomes, and that of all mapped genes, for each of the trios (named after the focal lineage under study), using modENCODE data obtained from 4 day old mated males. X->Auto refers to genes that moved out of the X, Auto->X refers to genes that moved into the X, Auto->Auto refers to genes that moved between autosomes, and All genes refers to all mapped genes in each trio. Significance was assessed with Wilcoxon tests (* for p<0.05, ** for p<0.01, *** for p<0.001, ns for non significant).

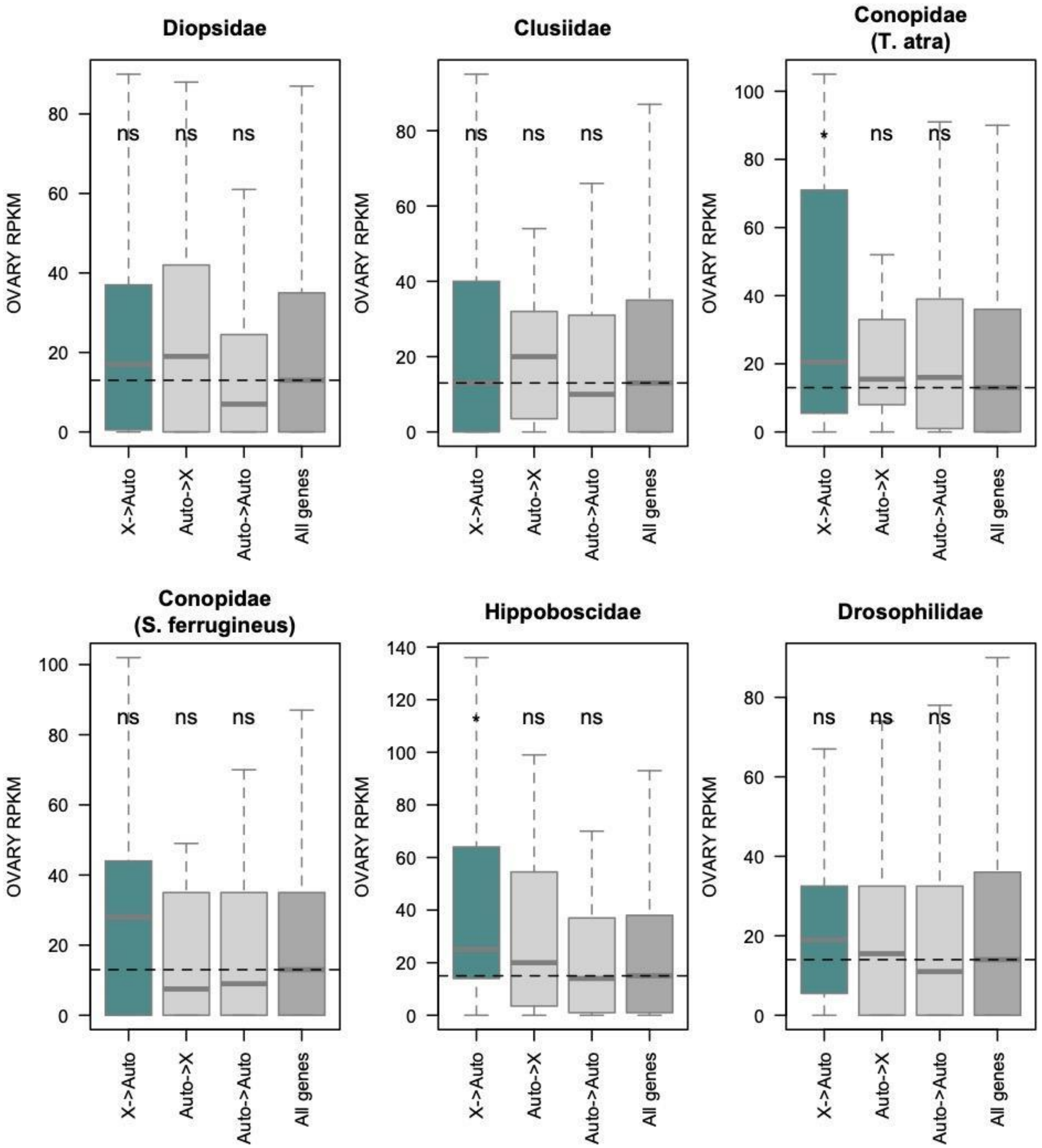

Figure S10: *D. melanogaster* ovary expression of homologs inferred to have moved chromosomes, and that of all mapped genes, for each of the trios (named after the focal lineage under study), using modENCODE data obtained from 4 day old mated females. X->Auto refers to genes that moved out of the X, Auto->X refers to genes that moved into the X, Auto->Auto refers to genes that moved between autosomes, and All genes refers to all mapped genes in each trio. Significance was assessed with Wilcoxon tests (* for p<0.05, ** for p<0.01, *** for p<0.001, ns for non significant).

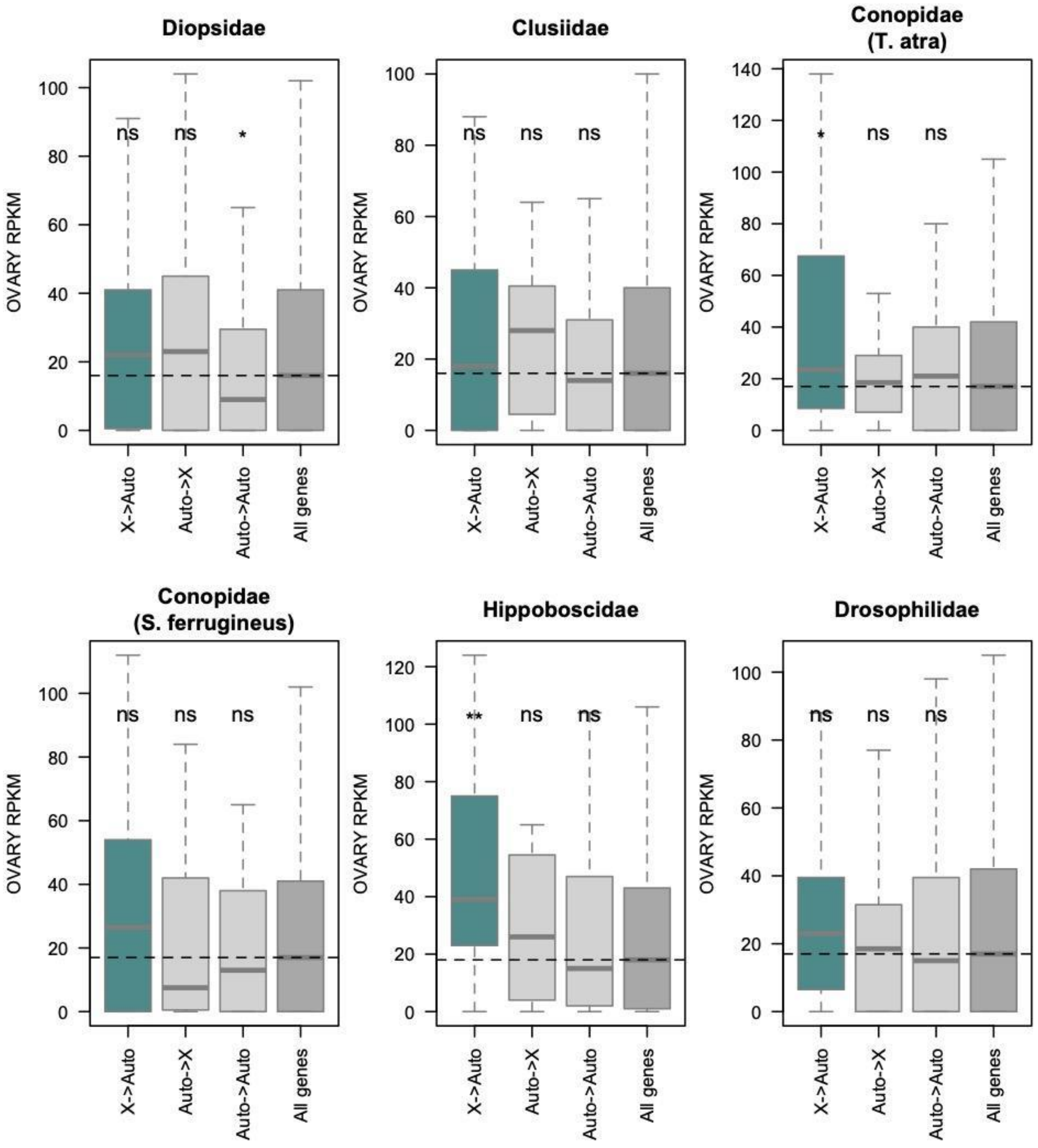

Figure S11: *D. melanogaster* ovary expression of homologs inferred to have moved chromosomes, and that of all mapped genes, for each of the trios (named after the focal lineage under study), using modENCODE data obtained from 4 day old virgin females**.** X->Auto refers to genes that moved out of the X, Auto->X refers to genes that moved into the X, Auto->Auto refers to genes that moved between autosomes, and All genes refers to all mapped genes in each trio. Significance was assessed with Wilcoxon tests (* for p<0.05, ** for p<0.01, *** for p<0.001, ns for non significant).

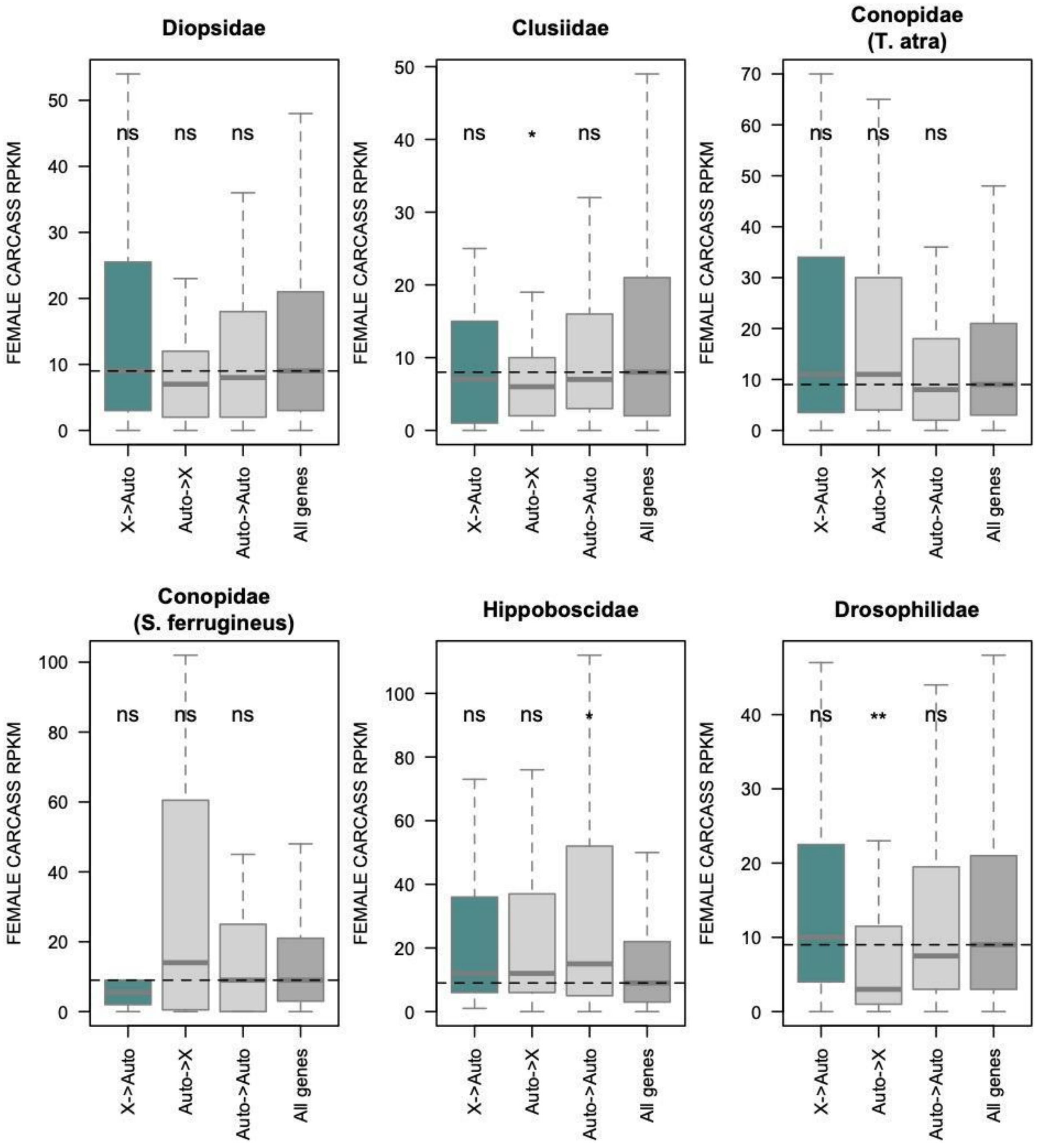

Figure S12: *D. melanogaster* female carcass expression of homologs inferred to have moved chromosomes, and that of all mapped genes, for each of the trios (named after the focal lineage under study). X->Auto refers to genes that moved out of the X, Auto->X refers to genes that moved into the X, Auto->Auto refers to genes that moved between autosomes, and All genes refers to all mapped genes in each trio. Significance was assessed with Wilcoxon tests (* for $p<0.05$, ** for $p<0.01$, *** for $p<0.001$, ns for non significant).

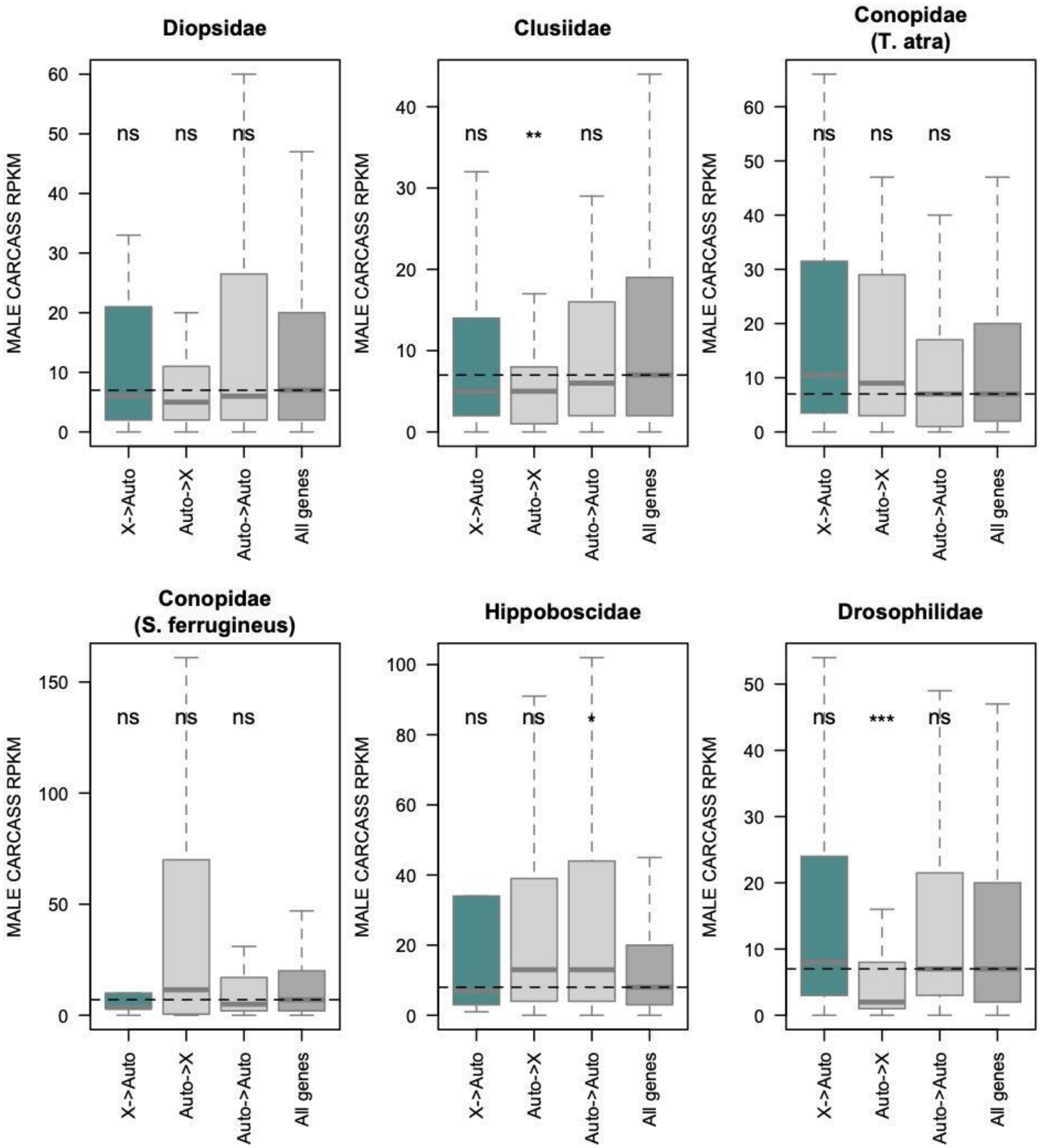

Figure S13: *D. melanogaster* male carcass expression of homologs inferred to have moved chromosomes, and that of all mapped genes, for each of the trios (named after the focal lineage under study). X->Auto refers to genes that moved out of the X, Auto->X refers to genes that moved into the X, Auto->Auto refers to genes that moved between autosomes, and All genes refers to all mapped genes in each trio. Significance was assessed with Wilcoxon tests (* for p<0.05, ** for p<0.01, *** for p<0.001, ns for non significant).

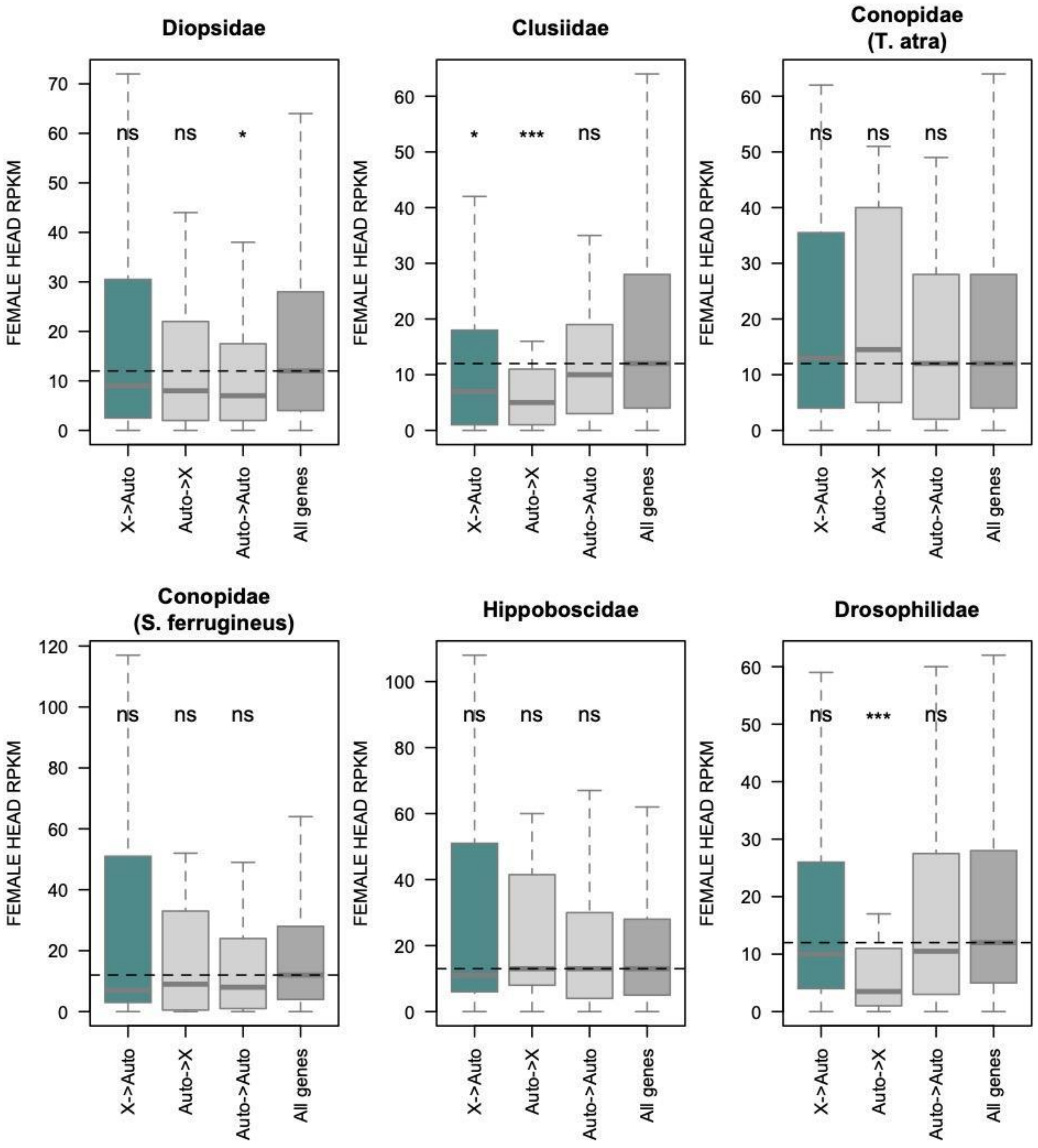

Figure S14: *D. melanogaster* female head expression of homologs inferred to have moved chromosomes, and that of all mapped genes, for each of the trios (named after the focal lineage under study). X->Auto refers to genes that moved out of the X, Auto->X refers to genes that moved into the X, Auto->Auto refers to genes that moved between autosomes, and All genes refers to all mapped genes in each trio. Significance was assessed with Wilcoxon tests (* for $p<0.05$, ** for $p<0.01$, *** for $p<0.001$, ns for non significant).

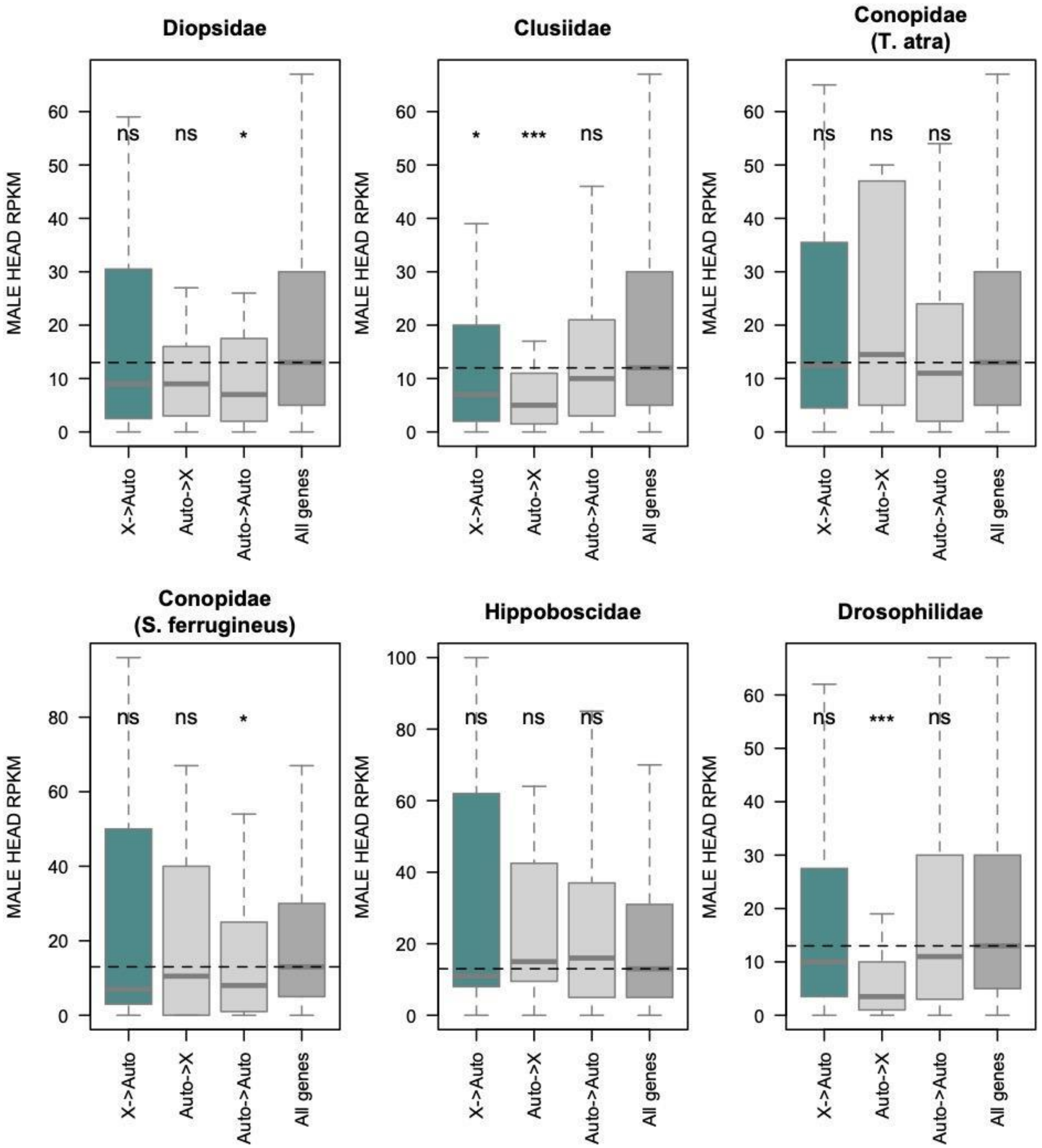

Figure S15: *D. melanogaster* male head expression of homologs inferred to have moved chromosomes, and that of all mapped genes, for each of the trios (named after the focal lineage under study). X->Auto refers to genes that moved out of the X, Auto->X refers to genes that moved into the X, Auto->Auto refers to genes that moved between autosomes, and All genes refers to all mapped genes in each trio. Significance was assessed with Wilcoxon tests (* for p<0.05, ** for p<0.01, *** for p<0.001, ns for non significant).

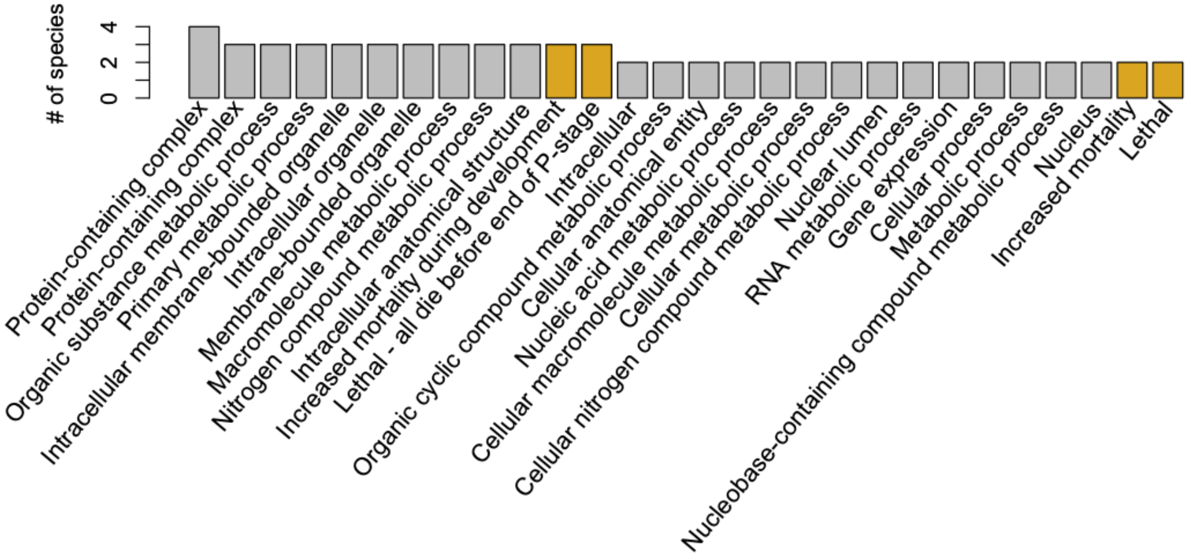

Figure S16: StringDB functional enrichment of genes that moved out of the X. The barplot shows the gene ontology (grey) and phenotype (yellow) terms that were enriched among genes that moved out of the X in at least 2 species, and the number of species this enrichment was found in.

Supplementary Tables

Table S3: The proportion of mapped genes found on the chromosome homologous to element F in species chosen to represent each of the families (shown in Figure 2). Lineages where element F is fused to another chromosome were excluded.

**Species**	**Family**	**% of genes on F-homologue**
Pegoplata infirma	Anthomyiidae	0.79%
Dysmachus trigonus	Asilidae	2.73%
Bibio marce	Bibionidae	3.18%
Bellardia pandia	Calliphoridae	0.66%
Clusia tigrina	Clusiidae	1.15%
Coelopa pilipes	Coelopidae	1.23%
Conops quadrifasciatus	Conopidae	1.21%
Dolichopus griseipennis	Dolichopodidae	1.73%
Drosophila pseudoobscura	Drosophilidae	0.70%
Dryomyza anilis	Dryomyzidae	1.02%
Rhamphomyia laevipes	Empididae	1.62%
Suillia variegata	Heleomyzidae	0.93%
Ornithomya chloropus	Hippoboscidae	0.60%
Hybos culiciformis	Hybotidae	1.45%
Calliopum simillimum	Lauxaniidae	1.03%
Toxonevra muliebris	Pallopteridae	0.90%
Nephrocerus scutellatus	Pipunculidae	1.94%
Pollenia amentaria	Polleniidae	0.68%
Ptychoptera albimana	Ptychopteridae	3.19%
Rhagio lineola	Rhagionidae	3.15%
Melanophora roralis	Rhinophoridae	0.74%
Sarcophaga caerulescens	Sarcophagidae	0.74%
Coremacera marginata	Sciomyzidae	1.25%
Beris chalybata	Stratiomyidae	2.64%
Blera fallax	Syrphidae	1.87%
Cistogaster globosa	Tachinidae	0.80%
Anastrepha ludens	Tephritidae	1.01%

Thereva nobilitata	Therevidae	2.67%
Nephrotoma appendiculata	Tipulidae	1.69%

Table S4: Protein annotations used for our Diptera-branched tree.

Busco	Family	*Species*	Link to annotations
	Acroceridae	*Acrocera orbiculus*	https://ftp.ebi.ac.uk/pub/ensemblorganisms/Acrocera_orbiculus/GCA_947359355.1/ensembl/geneset/2023_05/
	Anthomyiidae	*Pegoplata infirma*	https://ftp.ebi.ac.uk/pub/ensemblorganisms/Pegoplata_infirma/GCA_963921195.1/braker/geneset/2024_05/
	Asilidae	*Machimus atricapillus*	https://ftp.ebi.ac.uk/pub/ensemblorganisms/Machimus_atricapillus/GCA_933228815.1/ensembl/geneset/2022_08/
	Bibionidae	*Bibio marci*	https://ftp.ebi.ac.uk/pub/ensemblorganisms/Bibio_marci/GCA_910594885.2/ensembl/geneset/2024_05/
	Bombyliidae	*Bombylius discolor*	https://ftp.ebi.ac.uk/pub/ensemblorganisms/Bombylius_discolor/GCA_939192795.1/ensembl/geneset/2022_07/
	Calliphoridae	*Bellardia pandia*	https://ftp.ebi.ac.uk/pub/ensemblorganisms/Bellardia_pandia/GCA_916048285.2/braker/geneset/2022_03/
	Clusiidae	*Clusia tigrina*	https://ftp.ebi.ac.uk/pub/ensemblorganisms/Clusia_tigrina/GCA_920105625.2/braker/geneset/2023_07/
BUSCO	Coelopidae	*Coelopa pilipes*	GCA_947389925.1
	Conopidae	*Thecophora atra*	https://ftp.ebi.ac.uk/pub/ensemblorganisms/Thecophora_atra/GCA_937620795.1/braker/geneset/2022_07/
	Culicidae	*Anopheles gambiae*	GCF_943734735.2
	Diopsidae	*Teleopsis dalmanni*	GCF_002237135.1
	Dolichopodidae	*Poecilobothrus nobilitatus*	https://ftp.ebi.ac.uk/pub/ensemblorganisms/Poecilobothrus_nobilitatus/GCA_947095535.1/ensembl/geneset/2024_05/
	Drosophilidae	*Drosophila melanogaster*	GCF_000001215.4
BUSCO	Dryomyzidae	*Dryomyza anilis*	GCA_951804985.1
	Empididae	*Empis livida*	https://ftp.ebi.ac.uk/pub/ensemblorganisms/Empis_livida/GCA_963932195.1/ensembl/geneset/2024_05/
	Heleomyzidae	*Suillia variegata*	https://ftp.ebi.ac.uk/pub/ensemblorganisms/Suillia_variegata/GCA_949127995.1/braker/geneset/2023_07/
BUSCO	Hippoboscidae	*Ornithomya fringillina*	GCA_963978525.1
	Hybotidae	*Hybos culiciformis*	https://ftp.ebi.ac.uk/pub/ensemblorganisms/Hybos_culic

			iformis/GCA_964007475.1/braker/geneset/2024_05/
	Lauxaniidae	*Calliopum simillimum*	https://ftp.ebi.ac.uk/pub/ensemblorganisms/Calliopum_simillimum/GCA_951812925.1/braker/geneset/2023_10/
	Muscidae	*Hydrotaea cyrtoneurina*	https://ftp.ebi.ac.uk/pub/ensemblorganisms/Hydrotaea_cyrtoneurina/GCA_958296145.1/ensembl/geneset/2024_05/
	Pallopteridae	*Toxonevra muliebris*	https://ftp.ebi.ac.uk/pub/ensemblorganisms/Toxonevra_muliebris/GCA_963691655.1/braker/geneset/2024_05/
	Pipunculidae	*Nephrocerus scutellatus*	https://ftp.ebi.ac.uk/pub/ensemblorganisms/Nephrocerus_scutellatus/GCA_947095585.1/ensembl/geneset/2024_04/
	Polleniidae	*Pollenia amentaria*	https://ftp.ebi.ac.uk/pub/ensemblorganisms/Pollenia_amentaria/GCA_943735925.1/ensembl/geneset/2022_08/
	Ptychopteridae	*Ptychoptera contaminata*	https://ftp.ebi.ac.uk/pub/ensemblorganisms/Ptychoptera_contaminata/GCA_963942525.1/ensembl/geneset/2024_05/
BUSCO	Rhagionidae	*Rhagio lineola*	GCA_964211845.1
	Rhinophoridae	*Phyto melanocephala*	https://ftp.ebi.ac.uk/pub/ensemblorganisms/Phyto_melanocephala/GCA_941918925.1/braker/geneset/2022_07/
	Sarcophagidae	*Sarcophaga caerulescens*	https://ftp.ebi.ac.uk/pub/ensemblorganisms/Sarcophaga_caerulescens/GCA_927399465.1/ensembl/geneset/2022_03/
	Sciaridae	*Pseudolycoriella hygida*	GCA_029228625.1
	Sciomyzidae	*Coremacera marginata*	https://ftp.ebi.ac.uk/pub/ensemblorganisms/Coremacera_marginata/GCA_914767935.1/ensembl/geneset/2021_12/
	Stratiomyidae	*Chorisops tibialis*	https://ftp.ebi.ac.uk/pub/ensemblorganisms/Chorisops_tibialis/GCA_963669355.1/braker/geneset/2024_05/
	Syrphidae	*Blera fallax*	https://ftp.ebi.ac.uk/pub/ensemblorganisms/Blera_fallax/GCA_946965025.1/ensembl/geneset/2024_04/
	Tachinidae	*Cistogaster globosa*	https://ftp.ebi.ac.uk/pub/ensemblorganisms/Cistogaster_globosa/GCA_937654795.1/braker/geneset/2022_07/
	Tephritidae	*Anastrepha ludens*	GCF_028408465.1
	Therevidae	*Thereva nobiliata*	https://ftp.ebi.ac.uk/pub/ensemblorganisms/Thereva_nobilitata/GCA_963855945.1/braker/geneset/2024_05/
	Tipulidae	*Nephrotoma appendiculata*	https://ftp.ebi.ac.uk/pub/ensemblorganisms/Nephrotoma_appendiculata/GCA_947310385.1/ensembl/geneset/

			2023_05/
	Sepsidae	*Orygma luctuosum*	https://ftp.ebi.ac.uk/pub/ensemblorganisms/Orygma_luctuosum/GCA_965218485.1/ensembl/geneset/2025_04/
	Panorpidae	*Panorpa germanica*	https://ftp.ebi.ac.uk/pub/ensemblorganisms/Panorpa_germanica/GCA_963678705.1/
**Scaffolded assemblies:**			
BUSCO	Glossinidae	*Glossina fuscipes*	GCF_014805625.2
BUSCO	Ephydridae	*Cirrula hians*	
BUSCO	Agromyzidae	*Liriomyza trifolii*	GCA_043165165.1
BUSCO	Cecidomyiidae	*Sitodiplosis mosellanafa*	GCF_009176505.1
BUSCO	Scatopsidae	*Coboldia fuscipes*	GCA_001014335.1
BUSCO	Trichoceridae	*Trichoceridae species*	GCA_001014425.1

Table S7: GO enrichment amount genes that moved out of the X in Diopsidae. Rows highlighted in yellow show GO terms that remain enriched when mapped genes are used as the background to test against.

**#category**	**term ID**	**term description**	**observed gene count**	**false discovery rate**
GO Component	GO:0032991	Protein-containing complex	30	0.0019
GO Component	GO:0005622	Intracellular anatomical structure	48	0.003
GO Component	GO:0043226	Organelle	43	0.005
GO Component	GO:0043227	Membrane-bounded organelle	40	0.0058
GO Component	GO:0140535	Intracellular protein-containing complex	11	0.0066
GO Component	GO:0005665	RNA polymerase II, core complex	3	0.0095
GO Component	GO:0005634	Nucleus	27	0.0106
GO Component	GO:0043229	Intracellular organelle	41	0.0106
GO Component	GO:0070013	Intracellular organelle lumen	14	0.0153
GO Component	GO:0043231	Intracellular membrane-bounded organelle	37	0.0168
GO Component	GO:0031981	Nuclear lumen	12	0.0195
GO Component	GO:0005929	Cilium	6	0.0202
GO Component	GO:1990234	Transferase complex	9	0.0305
GO Component	GO:0070776	MOZ/MORF histone acetyltransferase complex	2	0.0317
**KEGG**	**dme03020**	**RNA polymerase**	**4**	**0.0019**

Reactome	DME-6781823	Formation of TC-NER Pre-Incision Complex	5	0.0048
Reactome	DME-6782210	Gap-filling DNA repair synthesis and ligation in TC-NER	4	0.0342
Reactome	DME-6782135	Dual incision in TC-NER	4	0.0343
Reactome	DME-3700989	Transcriptional Regulation by TP53	6	0.0465
WikiPathways	WP177	Eukaryotic transcription initiation	3	0.0131
WikiPathways	WP406	Mitochondrial long chain fatty acid beta-oxidation	2	0.015
COMPARTMENTS	GOCC:0005665	RNA polymerase II, core complex	3	0.0203
COMPARTMENTS	GOCC:0005929	Cilium	6	0.0203
COMPARTMENTS	GOCC:0031981	Nuclear lumen	12	0.0203
COMPARTMENTS	GOCC:0032991	Protein-containing complex	28	0.0203
COMPARTMENTS	GOCC:0043226	Organelle	34	0.0203
COMPARTMENTS	GOCC:0070013	Intracellular organelle lumen	14	0.0203
COMPARTMENTS	GOCC:0043229	Intracellular organelle	32	0.0283
COMPARTMENTS	GOCC:0005622	Intracellular	37	0.0313
UniProt Keywords	KW-0539	Nucleus	17	0.0068
UniProt Keywords	KW-0240	DNA-directed RNA polymerase	3	0.0218
InterPro	IPR036603	RNA polymerase, RBP11-like subunit	3	0.0122

Table S8: GO enrichment amount genes that moved out of the X in Clusiidae.

#category	term ID	term description	observed gene count	false discovery rate
GO Process	GO:0006807	Nitrogen compound metabolic process	29	0.0072
GO Process	GO:0010467	Gene expression	16	0.0072
GO Process	GO:0043170	Macromolecule metabolic process	27	0.0072
GO Process	GO:0044238	Primary metabolic process	30	0.0072
GO Process	GO:0071704	Organic substance metabolic process	32	0.0072
GO Process	GO:1901360	Organic cyclic compound metabolic process	17	0.0199
GO Process	GO:0006725	Cellular aromatic compound metabolic process	16	0.043
GO Component	GO:0032991	Protein-containing complex	23	0.0408
Monarch	FBcv:0002000	Lethal - all die before end of P-stage	24	0.0061
Monarch	FBcv:0002019	Increased mortality during development	31	0.0288
COMPARTMENTS	GOCC:0032991	Protein-containing complex	26	0.0017

Table S9: GO enrichment amount genes that moved out of the X in Conopidae (*Thecophora atra*).

#category	term ID	term description	observed gene count	false discovery rate
GO Process	GO:0044237	Cellular metabolic process	29	0.00054
GO Process	GO:0008152	Metabolic process	31	0.0027
GO Process	GO:0043170	Macromolecule metabolic process	26	0.0027
GO Process	GO:0044260	Cellular macromolecule metabolic process	17	0.0027
GO Process	GO:0090304	Nucleic acid metabolic process	15	0.0027
GO Process	GO:0006807	Nitrogen compound metabolic process	27	0.0036
GO Process	GO:0009987	Cellular process	39	0.0036
GO Process	GO:0006139	Nucleobase-containing compound metabolic process	16	0.0043
GO Process	GO:0034641	Cellular nitrogen compound metabolic process	18	0.0064
GO Process	GO:0071704	Organic substance metabolic process	28	0.0112
GO Process	GO:0044085	Cellular component biogenesis	15	0.0114
GO Process	GO:0044238	Primary metabolic process	27	0.0114
GO Process	GO:0071840	Cellular component organization or biogenesis	22	0.014
GO Process	GO:0016571	Histone methylation	4	0.0155
GO Process	GO:0022613	Ribonucleoprotein complex biogenesis	7	0.0183
GO Process	GO:0016070	RNA metabolic process	11	0.0276

GO Process	GO:0042254	Ribosome biogenesis	6	0.0276
GO Component	GO:0005622	Intracellular anatomical structure	39	8.84E-05
GO Component	GO:0043229	Intracellular organelle	34	0.0011
GO Component	GO:0043227	Membrane-bounded organelle	31	0.0045
GO Component	GO:0043231	Intracellular membrane-bounded organelle	30	0.0058
GO Component	GO:0005634	Nucleus	21	0.0164
GO Component	GO:0032991	Protein-containing complex	21	0.0164
GO Component	GO:0031981	Nuclear lumen	10	0.0255
GO Component	GO:0110165	Cellular anatomical entity	41	0.0255
GO Component	GO:0035097	Histone methyltransferase complex	3	0.0462
Reactome	DME-6791226	Major pathway of rRNA processing in the nucleolus and cytosol	5	0.013
Monarch	FBcv:0000351	Lethal	24	0.0383
Monarch	FBcv:0000379	Enhancer of variegation	3	0.0383
Monarch	FBcv:0000425	Increased cell death	8	0.0383
Monarch	FBcv:0002000	Lethal - all die before end of P-stage	20	0.0383
Monarch	FBcv:0002004	Increased mortality	30	0.0383
Monarch	FBcv:0002019	Increased mortality during development	29	0.0383

Monarch	FBcv:0002027	Lethal - all die before end of pupal stage	19	0.0383
UniProt Keywords	KW-0853	WD repeat	6	0.0074
SMART	SM00320	WD40 repeats	6	0.0207

Table S10: GO enrichment amount genes that moved out of the X in Drosophilidae. Rows highlighted in yellow show GO terms that remain enriched when mapped genes are used as the background to test against.

#category	term ID	term description	observed gene count	false discovery rate
**GO Process**	**GO:0006139**	**Nucleobase-containing compound metabolic process**	**33**	**1.69E-05**
**GO Process**	**GO:0034641**	**Cellular nitrogen compound metabolic process**	**38**	**1.69E-05**
GO Process	GO:0044237	Cellular metabolic process	57	1.69E-05
**GO Process**	**GO:0090304**	**Nucleic acid metabolic process**	**30**	**1.69E-05**
**GO Process**	**GO:1901360**	**Organic cyclic compound metabolic process**	**34**	**3.80E-05**
GO Process	GO:0008152	Metabolic process	62	0.00021
GO Process	GO:0044238	Primary metabolic process	56	0.00083
GO Process	GO:0071704	Organic substance metabolic process	57	0.0022
GO Process	GO:0043170	Macromolecule metabolic process	47	0.0023
GO Process	GO:0009987	Cellular process	81	0.0029
GO Process	GO:0006807	Nitrogen compound metabolic process	51	0.003
GO Process	GO:0006259	DNA metabolic process	12	0.0056
GO Process	GO:0006974	Cellular response to DNA damage stimulus	10	0.0091
GO Process	GO:0010467	Gene expression	23	0.0091

GO Process	GO:0044260	Cellular macromolecule metabolic process	26	0.0091
GO Process	GO:0006281	DNA repair	9	0.0107
GO Process	GO:0016070	RNA metabolic process	19	0.0138
GO Process	GO:0033554	Cellular response to stress	14	0.0424
GO Component	GO:0005622	Intracellular anatomical structure	84	3.16E-07
GO Component	GO:0043229	Intracellular organelle	73	6.60E-06
GO Component	GO:0043227	Membrane-bounded organelle	68	1.40E-05
GO Component	GO:0043231	Intracellular membrane-bounded organelle	66	1.81E-05
GO Component	GO:0032991	Protein-containing complex	46	4.25E-05
GO Component	GO:0005737	Cytoplasm	54	0.0187
GO Component	GO:0110165	Cellular anatomical entity	90	0.0187
Monarch	FBcv:0002004	Increased mortality	73	2.97E-06
Monarch	FBcv:0002019	Increased mortality during development	69	5.68E-06
Monarch	FBcv:0000351	Lethal	57	0.00012
Monarch	FBcv:0000354	Visible	54	0.00012
Monarch	FBcv:0002015	Partially lethal	39	0.0071
Monarch	FBcv:0000435	Abnormal neuroanatomy	28	0.0072
Monarch	FBcv:0002010	Majority die during P-stage	18	0.0072
Monarch	FBcv:0002005	Lethal - all die during P-stage	17	0.0073
Monarch	FBcv:0002000	Lethal - all die before end of P-stage	39	0.0143

Monarch	FBcv:0002020	Some die during P-stage	37	0.0143
Monarch	FBcv:0002007	Lethal - all die during larval stage	11	0.0197
Monarch	FBcv:0000352	Partially lethal - majority die	28	0.0441
Monarch	FBcv:0002039	Some die during pupal stage	30	0.0441
COMPARTMENTS	GOCC:0032991	Protein-containing complex	48	0.00011
COMPARTMENTS	GOCC:0005622	Intracellular	62	0.004

Table S11: GO enrichment amount genes that moved between autosomes in Hippoboscidae.

#category	term ID	term description	observed gene count	false discovery rate
GO Process	GO:0009950	Dorsal/ventral axis specification	6	0.0157
UniProt Keywords	KW-0995	Kinetochore	3	0.016
UniProt Keywords	KW-0720	Serine protease	6	0.0217
UniProt Keywords	KW-0137	Centromere	3	0.022
UniProt Keywords	KW-0645	Protease	8	0.0396

Table S12: GO enrichment amount genes that moved between autosomes in Drosophilidae.

#category	term ID	term description	observed gene count	false discovery rate
GO Component	GO:0032991	Protein-containing complex	57	0.00062
GO Component	GO:0043226	Organelle	90	0.00067
GO Component	GO:0005622	Intracellular anatomical structure	98	0.0027
GO Component	GO:0022627	Cytosolic small ribosomal subunit	6	0.0029
GO Component	GO:0043229	Intracellular organelle	86	0.0029
GO Component	GO:0110165	Cellular anatomical entity	123	0.0029
GO Component	GO:0015935	Small ribosomal subunit	7	0.0036
GO Component	GO:0043227	Membrane-bounded organelle	78	0.012
GO Component	GO:0022626	Cytosolic ribosome	7	0.0161
GO Component	GO:0044391	Ribosomal subunit	9	0.0172
GO Component	GO:0140513	Nuclear protein-containing complex	22	0.0205
Reactome	DME-72695	Formation of the ternary complex, and subsequently, the 43S complex	7	0.0023
Reactome	DME-72649	Translation initiation complex formation	7	0.0036
Reactome	DME-72702	Ribosomal scanning and start codon recognition	7	0.0036
Reactome	DME-8953854	Metabolism of RNA	17	0.0042
Reactome	DME-72689	Formation of a pool of free 40S subunits	7	0.0166

Reactome	DME-156827	L13a-mediated translational silencing of Ceruloplasmin expression	7	0.0333
Reactome	DME-72706	GTP hydrolysis and joining of the 60S ribosomal subunit	6	0.041
Reactome	DME-1799339	SRP-dependent cotranslational protein targeting to membrane	6	0.0411
Reactome	DME-975956	Nonsense Mediated Decay (NMD) independent of the Exon Junction Complex (EJC)	6	0.0425
Monarch	FBcv:0000443	Minute	6	0.00068
COMPARTMENTS	GOCC:0015935	Small ribosomal subunit	7	0.0109
COMPARTMENTS	GOCC:0022627	Cytosolic small ribosomal subunit	6	0.0109
COMPARTMENTS	GOCC:0032991	Protein-containing complex	54	0.0109
